# *O*-GlcNAc profiling: from proteins to proteomes

**DOI:** 10.1186/1559-0275-11-8

**Published:** 2014-03-05

**Authors:** Junfeng Ma, Gerald W Hart

**Affiliations:** 1 Department of Biological Chemistry, The Johns Hopkins University School of Medicine, 725 North Wolfe Street, Baltimore, MD 21205-2185, USA

**Keywords:** *O*-GlcNAc, *O*-GlcNAcome, *O*-GlcNAcomics, Proteomics, Enrichment, Site mapping, Quantification, Mass spectrometry

## Abstract

*O*-linked β-D-*N*-acetylglucosamine (*O*-GlcNAc) modification (*O*-GlcNAcylation) onto serine and threonine residues of proteins is an important post-translational modification (PTM), which is involved in many crucial biological processes including transcription, translation, proteasomal degradation, and signal transduction. Aberrant protein *O*-GlcNAcylation is directly linked to the pathological progression of chronic diseases including diabetes, cancer, and neurodegenerative disorders. Identification, site mapping, and quantification of *O*-GlcNAc proteins are a prerequisite to decipher their functions. In this review, we mainly focus on technological developments regarding *O*-GlcNAc protein profiling. Specifically, on one hand, we show how these techniques are being used for the comprehensive characterization of certain targeted proteins in which biologists are most interested. On the other hand, we present several newly developed approaches for *O*-GlcNAcomic profiling as well as how they provide us with a systems perspective to crosstalk amongst different PTMs and complicated biological events. Promising technical trends are also highlighted to evoke more efforts by diverse laboratories, which would further expand our understanding of the physiological and pathological roles of protein *O*-GlcNAcylation in chronic diseases.

## Background

Cellular proteins are often decorated by multiple post-translational modifications (PTMs) including glycosylation, phosphorylation, methylation, acetylation, and ubiquitylation (a detailed list of more than 400 different PTMs can be seen at http://www.uniprot.org/docs/ptmlist), which exert various biological functions in numerous processes. Among all the PTMs, glycosylation, which generally involves the covalent attachment of glycans to Ser/Thr/Asn residues, is predicted to occur in 80-90% of all extracellular and nucleocytoplasmic proteins and thus it is probably the most abundant and structurally diverse [1,2]. The classical glycosylation mainly occurs between Asn-linked (*N*-linked) or Ser/Thr-linked (‘mucin-type’ *O*-linked) oligosaccharides and cell surface and secreted proteins. However, *O*-linked β-D-*N*-acetylglucosamine modification (*O*-GlcNAcylation) is, 1) a monosaccharide modification onto hydroxyl groups of Ser/Thr residues, which is not elongated to complex sugar structures [3,4]; 2) almost exclusively on proteins localized in the nucleus, cytoplasm, and mitochondria [5]; 3) reversible and highly dynamic, which is controlled by two enzymes: *O*-GlcNAc transferase (OGT) (which catalyzes the addition of *O*-GlcNAc to Ser/Thr residues [6-8]) and β-D-*N*-acetylglucosaminidase (*O*-GlcNAcase) (which removes *O*-GlcNAc [9]); 4) interplayed with other PTMs (e.g., reciprocal/competitive with phosphorylation [10-12]); and 5) most common in metazoans.

Since its discovery in the early 1980s [3,4], *O*-GlcNAcylation has been found to play key roles in many fundamental biological processes including epigenetic regulation, transcription, translation, proteasomal degradation, signal transduction, stress response, and homeostasis, thus *O*-GlcNAc regulates diverse physiological events like circadian rhythm, memory formation, and learning [13-16]. Of particular note, since the synthesis of UDP-GlcNAc, the substrate donor for OGT, is tightly regulated by multiple major metabolic pathways in cells (i.e., glucose metabolism, amino acid metabolism, fatty acid metabolism and nucleotide metabolism) *via* the hexosamine biosynthesis pathway [17], *O*-GlcNAc is a sensitive nutrient sensor which links cellular metabolism with versatile signaling pathways. Therefore, it is not surprising that aberrant protein *O*-GlcNAcylation underlies the etiology and pathological progression of a number of chronic metabolic diseases including diabetes [18,19], cancer [20-22], and neurodegenerative disorders [23,24].

Even though numerous techniques have been developed during the last several decades to study *O*-GlcNAc (see excellent reviews [25-30]), there is still a strong demand for highly efficient tools, including methods for *O*-GlcNAc site mapping and production of site-specific antibodies. The development of facile and robust approaches for the assignment of the *O*-GlcNAc sites on proteins, a prerequisite for site-specific *O*-GlcNAc functional assays, would greatly facilitate probing the important roles of protein *O*-GlcNAcylation in various cellular processes. In this review, we mainly cover two aspects, 1) describe the classical and modern methods for the identification and site mapping of targeted *O*-GlcNAc proteins from a historic view, which would be helpful to biologists working on certain protein(s) and 2) delineate some newly developed enrichment and quantification techniques coupling with mass spectrometry (MS) for large-scale *O*-GlcNAc profiling from a proteomics view, which should offer a systems perspective for the function of *O*-GlcNAcylation on multiple proteins in physiology and diseases. Moreover, a discussion on future technology development for *O*-GlcNAc protein profiling is provided.

### Targeted *O*-GlcNAc protein profiling

As with other PTMs, *O*-GlcNAcylation of myriad proteins confers significant functions including alterations in protein stability and enzymatic activity, translocation (e.g., from the cytosol to the nucleus), and regulation of gene expression. Therefore, unambiguous identification of the *O*-GlcNAcylation status of protein(s) is of primary priority. Classical approaches, such as Western blotting and autoradiography, are still commonly used to confirm the existence of *O*-GlcNAc on targeted proteins.

Moreover, *O*-GlcNAcylation exerts diverse actions *via* a site-specific manner. Akin to phosphorylation, *O*-GlcNAcylation can occur on multiple Ser/Thr residues of proteins and *O*-GlcNAcylation on different sites often has distinct functional consequences. Therefore, the comprehensive characterization of all modification sites on proteins is a prerequisite to elucidate their roles. Biological mass spectrometry, a relatively newly emerging technique, has gained popularity for *O*-GlcNAc site assignment in recent years.

#### Classical biochemical assay for the identification of protein O-GlcNAcylation

#### UDP-[^3^H]-galactose labeling

Tritiated UDP-Galactose (i.e., UDP-[^3^H]-galactose)-based ‘hot labeling’ was used in the discovery of protein *O*-GlcNAcylation nearly 30 years ago [3,4] and is still a gold standard for determining protein *O*-GlcNAcylation status. In this approach, [^3^H]-galactose is added to the GlcNAc moiety on target proteins by β1-4-galactosyltransferase (GalT), allowing for detection by autoradiography. Another advantage is that by combining UDP-[^3^H]-galactose labeling with β-elimination and subsequent analysis of the released disaccharide product, the presence of a single GlcNAc residue can be confirmed. Of note, 1) proteins should be denatured for efficient incorporation of the galactose residues; and 2) since tritium is not as sensitive as other radiolabels, signals may take weeks to detect by autoradiography. Moreover, treatment with peptide: *N*-glycosidase F (PNGase F), a specific enzyme that removes nearly all *N*-linked glycans which may contain terminal GlcNAc residues, should be performed prior to UDP-[^3^H]-galactose labeling. In addition, nuclear/cytoplasmic extraction might be helpful to reduce the potential contamination of proteins from the endoplasmic reticulum/Golgi apparatus, the intracellular machinery for the synthesis of diverse types of glycans.

#### O-GlcNAc antibodies

The advent of a number of antibodies including CTD 110.6 [31], RL2 [32,33], and others [34-36] (see Table 1), which recognize the GlcNAc moiety on proteins, greatly expands the tools to probe *O*-GlcNAcylated proteins and enables Western blotting a facile approach for detecting protein *O*-GlcNAcylation. In comparison to the classical UDP-[^3^H]-galactose labeling approach, blotting with *O*-GlcNAc antibodies is a much more sensitive and convenient tool. Notably, each of these antibodies is raised to a specific *O*-GlcNAc-dependent epitope and only recognizes a subset of *O*-GlcNAc–modified proteins (although CTD 110.6, which is relatively less dependent on the protein structure, recognizes a wider range of *O*-GlcNAcylated proteins). Due to the partial complementarity towards *O*-GlcNAc recognition between these antibodies, their combined use often benefits the detection of protein *O*-GlcNAcylation status. Thus, multiple immunoblotting analysis with several antibodies is recommended to determine whether the proteins of interest are modified by *O*-GlcNAc.

**Table 1:** **Recognition of antibodies and lectins toward *O*-GlcNAc (adapted from**[29]**; Russell Reeves and Natasha E. Zachara, personal communications)**

	**Reagent name**	**Antibody isotype**	**Specificity**			**Commercial availability**	**Ref.**
			***O*-GlcNAc**	**β-GlcNAc**	GlcNAc*		
Anti-body	CTD110.6	IgM		+		+	[31]
	RL2	IgG	+			+	[32,33]
	HGAC39	IgG		+			[34]
	HGAC49	IgM		+			[34]
	HGAC85	IgG		+		+	[34]
	9D1.E4(10)	IgG		+		+	[35]
	18B10.C7 (3)	IgG	+			+	[35]
	1 F5.D6(14)	IgG	+			+	[35]
	My95	IgG					[36]
Lectin	WGA/sWGA^+^				+	+	[38]
	GSLII				+	+	[39]

GlcNAc*: all terminal GlcNAc including α/β-GlcNAc.

sWGA^+^: succinated-wheat germ agglutinin. sWGA reduces its affinity for sialic acid and GlcNAc residues. As such, sWGA is typically used for immunoblotting while WGA for purification.

It should be borne in mind that cross-reactivity between *O*-GlcNAc and other sugars might occur when probing with antibodies [37]. To avoid false positives, several procedures can be included, 1) treatment with PNGase F, and 2) *O*-GlcNAc competitive assay (i.e., preincubation of the antibody with 0.1-1 M free GlcNAc before blotting to compete the signal away), and 3) hexosaminidase treatment as a negative control to exclude the ‘mucin-type’ *O*-linked glycosylation. With the combination of upstream immunoprecipitation (IP) from complex samples, antibody-based immunoblotting remains the common practice for the detection of protein *O*-GlcNAcylation. Moreover, by measuring the intensity (e.g., with densitometry) of the target bands, relative *O*-GlcNAc changes can be obtained from samples under different treatment conditions.

Although certain lectins (e.g., wheat germ agglutinin (WGA) [38] and griffonia simplicifolia lectin II (GSLII) [39]) also show some specificity to *O*-GlcNAc moieties, they are more frequently used as an enrichment tool instead, which will be discussed in more detail later in this review.

### Methods for O-GlcNAc site mapping

Even though the *O*-GlcNAc status of proteins can be confirmed by using antibodies and/or UDP-[^3^H]-galactose labeling followed by autoradiography, it is indispensible to know the exact modification sites if the detailed molecular functions of site-specific O-GlcNAcylation are desired. To this end, Edman sequencing and mass spectrometry (MS) are the two main techniques that have been adopted.

#### Edman sequencing

Edman sequencing, which was initially developed for peptide sequencing, has made great contributions to map *O*-GlcNAc sites, especially in the early days of *O*-GlcNAc research [40-43]. This approach is usually used in conjunction with the UDP-[^3^H]-galactose labeling and high performance liquid chromatography (HPLC). In general, several steps are involved, 1) purified *O*-GlcNAc proteins (e.g., *via* immunoprecipitation) are reacted with UDP-[^3^H]galactose in the presence of GalT, 2) the resulting [^3^H]-galactose-labeled proteins are digested (commonly by proteases), with the digests separated by HPLC, and 3) fractions with high liquid scintillation counting values (containing radioactive-labeled *O*-GlcNAc peptides) are subjected to manual or automated Edman sequencing. The *O*-GlcNAcylated amino acids can be recovered and further characterized. However, there are several issues to be addressed: 1) Since Edman degradation requires purified peptides for amino acid sequencing, the starting material should be a purified protein or simple mixtures so that there are no co-eluted peptides in the HPLC fractions (pre-fractionation means like SDS-PAGE should be carried out if the mixture is too complex for HPLC resolution); and 2) Due to substantial sample loss (largely due to multiple rounds of HPLC) and fairly low sensitivity, a minimum of 20 pmole of starting material (where >20% of the sample is *O*-GlcNAcylated) is generally required. These caveats may cause problems for the *O*-GlcNAc site mapping of low abundance endogenous proteins but it should be amenable to recombinant proteins or synthetic peptides when mass spectrometers are not available. This approach would be very useful for the differentiation of isobaric masses of *O*-GlcNAc modifications on peptides (e.g., an *O*-GlcNAc moiety could be localized at one of the several Ser/Thr residues in a peptide, while the peptide mass is the same), which is often problematic for even advanced mass spectrometers. In addition, the phenylthiohydantoin derivatives of Ser/Thr-GalNAc and Ser/Thr-GalNAc-Gal can be well separated with an Edman sequencer [40].

#### Mass spectrometry (MS)

In contrast to Edman sequencing, MS is a powerful analytical tool that enables obtaining accurate information of proteins/peptides (e.g., molecular weight, amino acid sequence, and even sample quantity). Indeed, researchers have fervently embraced almost every new instrumentation advance in MS for *O*-GlcNAc research. Fast atom bombardment mass spectrometry (FAB-MS), the first widespread instrument suitable for ionizing peptides devised in the 1980s [44], was adopted for site mapping OGT-labeled synthetic peptides in early 1990s [45]. Shortly after FAB, the advent of electrospray ionization (ESI) [46] and matrix-assisted laser desorption-ionization (MALDI) [47], two ionization methods which are capable of directly ionizing involatile and labile biomolecules, have revolutionized the characterization of proteins/peptides. In combination with new fragmentation techniques (e.g., collision induced dissociation (CID), high-energy collision dissociation (HCD) [48], and electron transfer dissociation (ETD) [49] and several mass analyzers (e.g., time of flight (TOF), ion tap, and Orbitrap), the ESI/MALDI-based biological mass spectrometers provide tremendous impetus to the study of biomedical sciences, including the profiling of *O*-GlcNAcylated proteins. Moreover, the evolution of mass spectrometry has helped analyzing *O*-GlcNAc in a high throughput way. Undoubtedly, these advanced mass spectrometry techniques remain to be the cornerstone tools to date, due to the high sensitivity, selectivity, and throughput.

#### Electrospray ionization-collision induced dissociation-tandem mass spectrometry (ESI-CID-MS/MS)

**Figure 1:**
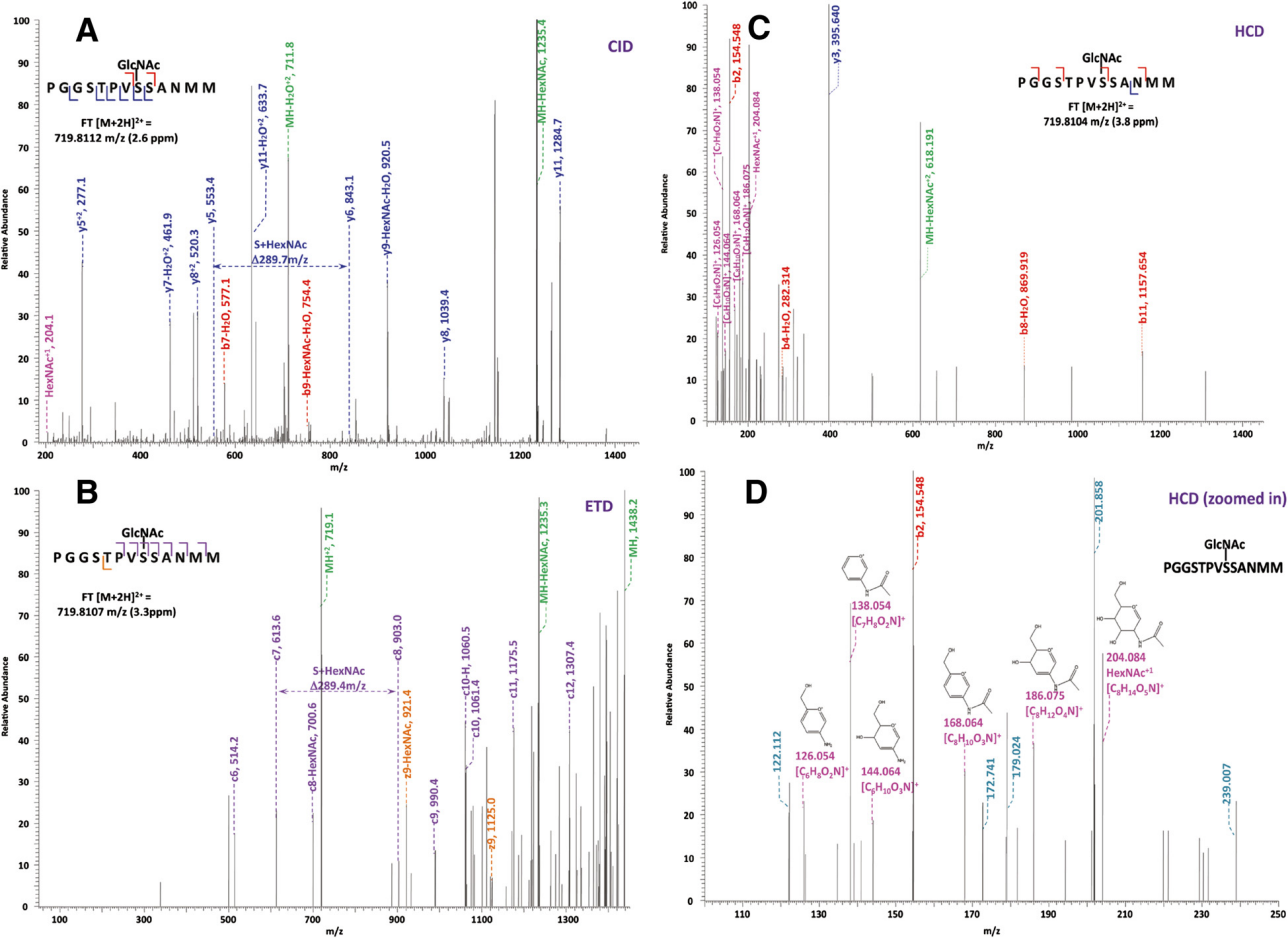
**Respective spectra from CID (A), ETD (B), HCD (C), and zoomed in HCD (D) of standard *O*-GlcNAc modified peptide CKII (PGGSTPV *s *SANMM, where ‘*s*’ represents the *O*-GlcNAc modified Ser).** Note: “-HexNAc” or “-H2O” indicates the loss of HexNAc or H2O. Low m/z range HCD displays a distinctive pattern of HexNAc fragments (D). (Adapted from [53], with the permission from American Chemical Society)

Relative to matrix-assisted laser desorption ionization-time of flight-tandem mass spectrometry (MALDI-TOF-MS/MS), ESI-CID-MS/MS has gained enormous popularity for its almost perfect demonstration of characterizing many types of PTMs on proteins/peptides. However, regarding *O*-GlcNAc site mapping, limited success has been achieved. For example, the electrospray ionization-collision induced dissociation-quadrupole time-of-flight tandem mass spectrometry (ESI-CID-Q-TOF-MS/MS) has been used for direct identification of *O*-GlcNAc sites on synthetic peptides [50] and from in-gel digests of over-expressed serum response factor [51]. A major challenge for direct detection by ESI-CID-MS/MS is that the glycosidic bond between *O*-GlcNAc and its peptide sequence is more susceptible to breakage than that of the peptide backbone during CID, where relatively high collision energy is often applied. Therefore, the *O*-GlcNAc group is preferentially lost (producing an *O*-GlcNAc oxonium ion) prior to peptide fragmentation and thus, the exact modification sites can not be assigned. However, in some cases when large amounts of material are available, a very low percentage of fragment ions may still bear the *O*-GlcNAc moiety and may be useful in identifying modification sites (as exemplified in Figure 1) [52,53].

It is noteworthy that in comparison to conventional CID, the newly developed HCD fragmentation can produce and monitor the *O*-GlcNAc oxonium ion (+204.08) in a more efficient way (Figure 1) [53]. Not only that, a series of fragments of the *O*-GlcNAc oxonium ion (i.e., m/z 186.07, m/z 168.06, m/z 144.06, m/z 138.05, and m/z 126.05) can also be generated at pronounced high intensity. One striking benefit of the *O*-GlcNAc oxonium ion and its fragments is that they can serve as diagnostic ions for the presence of *O*-GlcNAc on certain peptides, although it would be difficult to accurately designate sites by using CID or HCD alone especially when there are more than one Ser/Thr residues in the peptide sequence. Another feature is that CID or HCD is adaptable to coupling with ETD (i.e., CID/ETD-MS/MS or HCD/ETD-MS/MS), enabling more reliable identification and site mapping of *O*-GlcNAc peptides *via* the alternating scanning mode or CID/HCD-triggered ETD mode.

One way to take advantage of the currently prevalent CID/HCD-MS/MS is to convert the labile glycosidic bond to a CID/HCD-compatible bond which can withstand the CID or HCD fragmentation. For example, alkaline-induced β-elimination can convert the *O*-GlcNAcylated Ser or Thr to 2-aminopropenoic acid and 2-amino-2-butenoic acid, respectively [54], or further to sulfide derivatives in the presence of reducing reagents (e.g., Dithiothreitol) [55].

#### Electrospray ionization-electron transfer dissociation-tandem mass spectrometry (ESI-ETD-MS/MS)

A very recent breakthrough in MS is the invention of ETD fragmentation technique [49]. Different from CID, ETD induces cleavage of the backbone N-Cα bond, generating c- and z-ions for peptide sequencing. More importantly, ETD generally does not break the linkage between PTMs and their modified residues, thus CID-labile PTMs can be well preserved during ETD (Figure 1), providing specific site information [56]. Therefore, the ESI-ETD-MS/MS method has been increasingly adopted, largely facilitating the direct site assignment of *O*-GlcNAcylated proteins (as exemplified in [57-59]).

Of note, a combination of several fragmentation approaches is very helpful for in-depth characterization since ETD tends to perform better than CID or HCD on higher charge states (Z>2 positive charges) but yields a lower number of total identifications due to its relatively slower scan rate and lower fragmentation efficiency [60]. Indeed, both CID/ETD-MS/MS [35,37,57,59] and HCD/ETD-MS/MS [53] have increased the confidence of *O*-GlcNAcylated peptide identification and site localization. In addition, pulsed Q dissociation (PQD) has also been coupled with ETD for a two-stage tandem MS approach for *O*-GlcNAc peptide analysis, facilitating the detection of such peptides by PQD at low collision energy and the identification and site localization by ETD [61]. By integrating with OScore [61], a scoring scheme which can discriminate *O*-GlcNAc peptide spectra from those of naked peptides with >99% specificity, *O*-GlcNAc peptides in the low fmole range were detected and a 10-fold higher sensitivity than a single data-dependent ETD-MS/MS experiment was achieved.

Although site-specific detection for *O*-GlcNAc peptides with ETD has gained great success, its utility for the *O*-GlcNAc site mapping on intact individual protein(s) (i.e., the ‘top-down’ approach) is still to be explored. Since ETD is also regarded to be advantageous for the fragmentation of longer peptides or even entire proteins [56], its application to *O*-GlcNAc analysis should be feasible at least for small individual proteins, which would provide another unique avenue for *O*-GlcNAc profiling.

#### *Special considerations for O*-GlcNAc *sample preparation*

To fulfill successful profiling of *O*-GlcNAc proteins of interest, several aspects concerning *O*-GlcNAc sample preparation should be considered beforehand, 1) starting amount of proteins/peptides, 2) efficient digestion of proteins, and 3) possible derivatization of *O*-GlcNAc peptides (especially for ETD detection).

Immunoprecipitation (IP) is commonly used to purify proteins of interest. However, for some biologically important proteins (e.g. transcription factors), which are often of low abundance, IP may not be practical to obtain enough material for the profiling of even lower abundant *O*-GlcNAcylated populations. In vitro OGT-labeling of recombinant proteins (or even synthetic peptides) followed by MS should be informative on potential *O*-GlcNAc modification sites. Another alternative approach involves the co-expression of OGT in the presence of the target protein (e.g., transcription factor Sp1 [62] and Tau [52]) in *E. Coli*. In this approach, OGT can somehow *O*-GlcNAcylate its substrates. No matter which method is adopted, further site-directed mutagenesis is necessary to verify the actual *O*-GlcNAc modification sites of the endogenous proteins in a given biological context.

Trypsin is often the preferred enzyme for protein digestion. However, in terms of mapping *O*-GlcNAc sites on certain proteins, adequate care should be taken. Although no consensus sequence for O-GlcNAc modification has been found so far, some *O*-GlcNAc proteins tend to have functionally important sites in sequences with clustered Ser/Thr residues and less or no arginine/lysine residues (e.g., ^374^DSSTDLTQTSSSGTVTLP^391^ in serum response factor [41], ^887^GFDT-SSSSSNSAASSSFK^904^ in Nucleoporin Nup153 [53], ^48^LSPP-SSSAASSYSFSDNLFTR^68^ in emerin [59], and tandem repeats of the consensus peptide sequence ‘SPTSPS’ in the C-terminal domain of RNA Polymerase II [42]). In such cases, digestion with trypsin is not always the best choice. Instead, proteolysis with chemical cleavage (e.g., with cyanogen bromide) and digestion with complimentary specificity enzymes (e.g., Asp-N, and Glu-C) could be beneficial for improved detection of certain long peptides with few or no tryptic sites as well as the assignment of their *O*-GlcNAc sites. Moreover, considering the limited success in the detection of low-charged peptides with ETD-MS/MS, derivatization to certain residues (e.g., to the C-terminal carboxyl group [63]) may also be performed to impart more positive charges and thus improved detection and site mapping of *O*-GlcNAc peptides.

#### O-GlcNAc site prediction

There are a number of bioinformatic software tools available for predicting modification sites for other PTMs [64]. However, only a few have been developed for *O*-GlcNAc prediction, namely, YinOYang [65], OGlcNAcScan [66], and *O*-GlcNAcPRED [67] (Table 2). With the analysis of an independent test dataset, *O*-GlcNAcPRED seems to have better performance than the other two predictors, especially in terms of prediction sensitivity. Providing the different computational models used, these tools also show certain complementarity in the prediction of *O*-GlcNAc sites. Undoubtedly, they may provide some useful reference for the modification status and potential *O*-GlcNAc sites as well as the experimental detection of *O*-GlcNAc on proteins of interest by MS. Further maturation of these tools (regarding the prediction accuracy and sensitivity) and the development of new ones would facilitate research on *O*-GlcNAcylation and proteomic identification.

**Table 2:** **Bioinformatic tools for *O*-GlcNAc site prediction**

***O*-GlcNAc predictor**	**Website**	**Algorithm models (Ref.)**
YinOYang	http://www.cbs.dtu.dk/services/YinOYang/	Artificial neuronal network [65]
dbOGAP (OGlcNAc Scan)	http://cbsb.lombardi.georgetown.edu/hulab/OGAP.html	Support vector machine [66]
*O*-GlcNAcPRED	(Not available yet)	Support vector machine [67]

### O-GlcNAc stoichiometry of proteins

Determination of *O*-GlcNAc stoichiometry of individual proteins provides additional information for understanding the function and regulation of protein *O*-GlcNAcylation. However, the addition of *O*-GlcNAc (+203) does not usually alter the apparent molecular weight of a protein (unlike the classical *N*-linked and *O*-linked glycoproteins), as judged by methods such as SDS-PAGE. Moreover, there are no changes in the charge status, leading to an unchanged pI value for a protein (which is different from phosphorylated proteins). Therefore, it is not feasible to distinguish the *O*-GlcNAc modified population from the naked one by SDS-PAGE itself. Recently, the development of a mass-tagging strategy shows strength in quantifying the *O*-GlcNAcylation level on specific proteins [68-71]. Basically, *O*-GlcNAcylated proteins are chemoenzymatically labeled using an UDP-ketogalactose analog and then reacted with an aminooxy-functionalized PEG mass tag (e.g., 5kDa). By doing so, the *O*-GlcNAcylated species will migrate differently than their native counterpart upon SDS-PAGE, which can be easily visualized by immunoblotting with antibodies against the protein of interest. The relative *O*-GlcNAcylation level can thus be determined by comparing the density of the modified species against that of the total population. The striking feature of this approach is that the *O*-GlcNAcylation state (e.g., mono-, di-, tri-) of proteins will be revealed if multiple bands as a ladder can be observed. One potential caveat is that incomplete labeling caused by either enzymatic or chemical reaction may also result in multiple bands. Therefore, further validation should be performed to confirm the multi-*O*-GlcNAcylation status to obtain accurate amount of each population for specific proteins.

### Global *O*-GlcNAcomic profiling

MS-based proteomics, a powerful technology referring to the analysis of the expression, localization, PTMs, and interactions of proteins expressed by a genome at a specific time, has greatly changed our view about intricate molecular networks [72-74]. By coupling high-resolution separation (mainly 2-D gel electrophoresis and HPLC) with unbiased isotopic labeling techniques, MS-based proteomics is capable of providing comprehensive characterization of thousands of proteins. Concomitantly, various enrichment methods toward specific PTMs have emerged, largely advancing the qualitative and quantitative analysis of PTM-proteomes including the *O*-GlcNAcome. Furthermore, protein microarrays have also been used for *O*-GlcNAcomic profiling.

#### Gel-based O-GlcNAcomics

Traditionally, 2-D gel electrophoresis separated spots are visualized with dyes, fluorophores, radioactivity, or antibody-based western blotting, enabling comparative analysis of proteins. The combined use of 2-D gel electrophoresis separation and MS detection, a core proteomic tool in the 1990s, has been applied to *O*-GlcNAc analysis in some studies [75-78]. Although certain success has been achieved, several issues which are closely related to the 2-D gel separation technique itself should be addressed [79], including 1) low efficiency in the analysis of hydrophobic or extremely acidic/basic proteins; 2) obscurity of low abundance proteins; 3) low quantitative accuracy due to the limited dynamic range; and 4) general unavailability of *O*-GlcNAc modification site information.

#### Gel-free O-GlcNAcomics

In comparison to 2-D gel electrophoresis, the gel-free separation approach (especially multiple dimensional HPLC for peptides) has catapulted MS-based proteomics (including PTM-proteomics) to an unprecedented level. As with other PTMs, *O*-GlcNAc proteins are generally regarded to be substoichiometric (e.g., less abundant than phosphorylation), although one study has shown that hundreds of *O*-GlcNAc peptides could be automatically identified from existing large-scale proteomic data sets with the recently developed software Oscore [61,80]. Moreover, there is severe ion suppression for detecting *O*-GlcNAc modified peptides in the presence of naked peptides [26]. In addition, as aforementioned, no consensus *O*-GlcNAc motif has been found yet. All the hurdles make accurate *O*-GlcNAc site assignment a challenging task. As with other PTMs, selective enrichment for *O*-GlcNAc is indispensable, especially when complex biological samples are to be analyzed.

According to the unique biochemical properties of *O*-GlcNAc, an array of enrichment techniques has been developed. With the aid of well-established quantification methods, large-scale *O*-GlcNAcomic profiling has begun to take off and contributed to a systems biological understanding of cells under physiological or pathological status.

#### Antibody based O-GlcNAc enrichment

High-affinity antibodies are generally the primary choice to pull down proteins/peptides with certain PTM(s). Although pan-specific antibodies (e.g., CTD 110.6, RL2) work well for *O*-GlcNAc immunoblotting, they have tentative applications for enriching *O*-GlcNAc proteins due to their relatively low affinity. By using CTD 110.6-conjugated beads enrichment and MS, Wang *et al.* identified 45 potentially *O*-GlcNAcylated proteins from COS7 cells [81]. With the combination of SILAC (i.e., stable isotope labeling with amino acids in cell culture), an apparent increase in *O*-GlcNAcylation of >10 proteins while a decreased *O*-GlcNAcylation of nearly 20 proteins was observed upon inhibition of glycogen synthase kinase-3 (GSK-3). With a similar approach, another study reported the identification of dozens of *O*-GlcNAc proteins from COS7 cells [82]. Among them, a number of proteins showed elevated levels of *O*-GlcNAcylation in response to heat stress.

While the production of higher-affinity *O*-GlcNAc antibodies seems extremely difficult, there has been a long-standing interest to develop novel antibodies over the years. The challenges of making *O*-GlcNAc antibodies mainly lie in two aspects: 1) *O*-GlcNAc–modified epitopes are often self-antigens that are tolerated by the immune system and 2) carbohydrate-protein interactions are relatively weak, which complicates antibody maturation [26,35]. Continuous efforts, however, have been made to generate *O*-GlcNAc antibodies that can be applied for immunocapture. Recently, with three *O*-GlcNAc-specific monoclonal antibodies [35] for the enrichment of *O*-GlcNAc proteins from HEK293 cell lysates, 83 *O*-GlcNAc sites were identified with HCD/ETD-MS/MS [53].

The combined usage of multiple antibodies and the development of higher-affinity antibodies should further improve the enrichment performance toward *O*-GlcNAc. One shortcoming with antibody-based *O*-GlcNAc protein enrichment is that proteins interacting with *O*-GlcNAcylated ones would also be pulled down, leading to false positive identification. Independent techniques (e.g., immunoblotting with CTD 110.6) should be used for confirmation. By combining this approach with an advanced mass spectrometer (e.g., ETD-MS/MS), the accurate modification sites on *O*-GlcNAcylated proteins can be identified, which would be a definitive indicator for the protein *O*-GlcNAcylation status.

#### Lectin based O-GlcNAc enrichment

Due to the binding interaction with the glycan structure on glycoconjugates, lectins serve as an important tool in glycoproteomics and glycomics [83]. However, only several lectins have been used for *O*-GlcNAc research so far.

Wheat germ agglutinin (WGA) is a lectin that recognizes both terminal GlcNAc and sialic acid residues. Although succinylated WGA (sWGA) increases the specificity to GlcNAc over sialic acid, its affinity toward GlcNAc is compromised as well [29]. Therefore, sWGA is mainly used for immunoblotting, although certain success in the capture of *O*-GlcNAc proteins has been shown in some cases. WGA, working as a dimer containing four carbohydrate binding sites, has high affinity interactions with complex glycans *via* multi-point binding [84]. Thus, it is not surprising that WGA shows a much lower affinity for the monomeric *O*-GlcNAc. Indeed, *O*-GlcNAc interaction with WGA is quite weak, as demonstrated by the ~10 mM dissociation constant for free GlcNAc to WGA [85]. In comparison to protein enrichment, *O*-GlcNAc peptide enrichment has gained much attention especially with the newly developed WGA-based lectin weak affinity chromatography (LWAC) technique [86-90]. In LWAC, conjugated WGA is packed into an adequately long column (e.g., 3 meters), which is then coupled downstream to a low flow rate isobaric HPLC instrument. By doing so, compared to the unmodified peptides, *O*-GlcNAc peptides are retarded by the column and recovered in later eluting fractions. The applicability of this strategy was first demonstrated through enrichment of 145 unique *O*-GlcNAc-modified peptides from a postsynaptic density (PSD) preparation [86]. By combining this enrichment approach with ETD-MS/MS, Chalkley *et al.* identified 58 modification sites from mouse PSD [87]. In a recent report, with the utilization of further optimized LWAC enrichment and peptide separation (i.e., offline fractionation via basic reversed phase high performance liquid chromatography (bRPLC)), 1750 *O*-GlcNAc sites were assigned to mouse brain synaptosomal proteins [89], greatly benefiting the future investigation of brain development and functions. In another study, with the combination of LWAC and SILAC for the analysis of the nuclear fraction from embryonic stem cells (ESCs), the same group unambiguously found 142 *O*-GlcNAc modification sites on 62 proteins, some of which are essential to maintain the ESC-specific expression profile [88]. Taken together, LWAC has shown reasonable affinity towards clustered *O*-GlcNAc-bearing peptides as well as singly and doubly *O*-GlcNAc-modified peptides. The success of this technique has greatly expanded the *O*-GlcNAc protein database. The more sophisticated application of such columns (e.g., improved collection of desired fractions to decrease loss of *O*-GlcNAc peptides) may promote a wider acceptance of this approach for the enrichment of *O*-GlcNAc peptides.

Besides WGA, another lectin Ricinus communis agglutinin I (RCA I) has been used for *O*-GlcNAc enrichment as well. However, different from WGA toward GlcNAc, RCA I can specifically recognize in vitro galactosylated GlcNAc. In this approach, GlcNAc-bearing peptides are incubated with UDP-galactose in the presence of GalT, with the resulting Galβ1-4-GlcNAc-peptides captured by conjugated RCA I. Compared to WGA for GlcNAc, RCA1 for Galβ1-4-GlcNAc (i.e., LacNAc) shows a higher affinity (Ka=10μM). Although several studies have used this approach for the enrichment of *O*-GlcNAc peptides from individual proteins [91-93] and improved binding specificity proposed, its feasibility for large-scale application has yet to be evaluated.

Collectively, lectins (especially WGA) are useful tools for the enrichment of *O*-GlcNAc peptides. To improve the binding specificity and capacity, PNGase F treatment is often required beforehand to remove *N*-linked GlcNAc-terminating sugars on proteins/peptides. Other lectins, which can improve the binding affinity to *O*-GlcNAc, are still worthy to be exploited for increased enrichment efficiency.

#### Chemical derivatization-based O-GlcNAc enrichment

Compared with antibody- and lectin-based *O*-GlcNAc enrichment, chemical derivatization is a large category of indirect enrichment, which is often comprised of three steps: derivatization, capture, and release. Specifically, the *O*-GlcNAc group is derivatized to add a handle (e.g., biotin) that can be readily captured onto beads (e.g., streptavidin-conjugated ones) and the released tagged *O*-GlcNAc peptides will then be subjected to MS detection. To date, several chemical-derivatization techniques have been developed for *O*-GlcNAc enrichment.

#### Hydrazide chemistry

Hydrazide chemistry is a well-established method for *N*-glycoproteomic profiling [94]. Recently, an appropriately modified analog has been developed for *O*-GlcNAc enrichment [95]. In this approach, several steps are involved: 1) a prolonged periodate oxidation is performed to convert the *O*-GlcNAc group to its dialdehyde derivative, 2) hydrazide resin is used to capture the oxidized *O*-GlcNAc peptides, and 3) after proteolytic digestion, the resulting modified peptides are released by hydroxylamine. With this enrichment procedure followed by MS/MS, several *O*-GlcNAc sites were identified from *Drosophila melanogaster* proteasome protein complex. To apply this technique for large-scale *O*-GlcNAc site mapping, two issues may need to be further addressed, 1) to derivatize the less active *O*-GlcNAc moiety (largely due to the trans configuration of the vicinal hydroxyls at positions C3 and C4), harsher conditions in periodate oxidation should be used, leading to undesired side reactions (e.g., oxidation of *N*-terminal Ser/Thr) and thus high background, and 2) more efficient and specific release of tagged *O*-GlcNAc peptides would be beneficial for the detection and site assignment of *O*-GlcNAc peptides.

#### β-elimination Michael addition (BEMA)

As aforementioned, *O*-GlcNAc can be removed from proteins/peptides by mild β-elimination, with Ser and Thr residues converted into their dehydrated equivalents (i.e., dehydroalanine and α-amino butyric acid, respectively) [54]. Based on this chemistry, a refined approach, named β-elimination Michael addition (BEMA), has been developed to mark the site of *O*-GlcNAc modification. In BEMA, the α/β-unsaturated carbonyl is derivatized with nucleophilic reagents (e.g., DTT or biotinylated pentylamine/cystamine) and the resulting peptides can then be enriched by thiol-capture resin or streptavidin-conjugated beads. Since DTT is the preferred nucleophile, the β-elimination Michael addition of DTT has been termed BEMAD [55,96]. There are several striking features of this method, 1) in comparison to the initial labile glycosidic bonds in *O*-GlcNAc peptides, the final resulting sulfide derivatives are stable enough during fragmentation and thus suitable for detection and site mapping by the most prevalent CID-MS/MS without relying on ETD technology and 2) quantitative *O*-GlcNAc site information can be readily achieved by using isotopically labeled DTT (i.e., D6-DTT and D10-DTT). Of special note is that, although phosphorylated peptides can also undergo BEMAD, faster conversion to the BEMAD product under milder conditions is achieved for *O*-GlcNAc peptides due to the more easily eliminated *O*-glycosidic linkages [3,54,55,96-98], with less undesired side reactions. Therefore, optimized BEMAD conditions and appropriate sample pre-treatment (e.g., with PNGase F) should be performed to avoid potential false positive identifications. In addition, distinct approaches (e.g., HCD-MS/MS or ETD-MS/MS, and immunoblotting) should be adopted for further validation. By using the BEMAD approach, several *O*-GlcNAc sites were determined from the key contractile proteins, such as actin and myosin heavy chains, in skeletal muscle [99]. Recently, an adapted method involving β-elimination-based derivatization with a biotin-cystamine tag followed by streptavidin-conjugated beads has been developed [100]. By differential isotopic labeling with either light biotin-cystamine or deuterated heavy biotin-cystamine, the specificity of the enrichment approach can be increased. Several *O*-GlcNAc sites within the murine 20*S* proteasome core complex were assigned.

The combined use of BEMAD and other techniques (e.g., chemoenzymatic labeling), which could further improve the enrichment specificity, is also favorable for *O*-GlcNAc profiling.

#### Chemoenzymatic labeling

Chemoenzymatic labeling capitalizes on the merits of the traditional GalT labeling and the advanced chemical derivatization techniques (especially the ketone-aminoxy process and bioorthogonal chemistry). Different from traditional GalT labeling, unnatural galactose analogues with specific chemical handles, which can facilitate the subsequent capture procedure, are used in chemoenzymatic labeling. An engineered mutant of GalT (GalT^Y289L^), which has an enlarged binding pocket for the donor-substrate [101], is the best choice to selectively derivatize *O*-GlcNAc with galactose analogues. To date, two major kinds of such analogues have been developed and used for chemoenzymatic labeling, i.e, ketone-bearing UDP-galactose and azido-modified UDP-galactose (UDP-GalNAz).

In one approach, GalT^Y289L^ is used to transfer the keto-galactose onto *O*-GlcNAc proteins and a biotin-aminoxy reagent is then attached *via* the oxime formation (aminoxylation). The biotin-tagged derivatives are visualized by streptavidin blotting [102] or subjected to streptavidin-conjugated beads enrichment followed by fluorescence [103] or MS detection [104,105]. By incorporating this approach with isotopic dimethyl labeling and ETD-MS/MS, comparative quantification of *O*-GlcNAc levels from two different brain populations was performed [105].

Another chemoenzymatic approach integrates UDP-GalNAz-based GalT^Y289L^ labeling, copper (I)-catalyzed azide-alkyne cycloaddition (click chemistry), and streptavidin-conjugated beads [106-110]. Since the biotin-streptavidin interaction is extraordinarily stable (Kd~= 10^-15^ M for the homo-tetramer streptavidin and 10^-7^-10^-8^ M for monomeric streptavidin), one elegant way is to introduce a UV-cleavable linker to afford improved release efficiency of tagged peptides from streptavidin-conjugated beads [107-109]. An additional advantage is that the released peptides contain a basic aminomethyltriazoyl acetylgalactosamine moiety, enabling efficient ETD fragmentation. By using the combination of GalT^Y289L^ labeling, click chemistry, UV-cleavage, and ETD-MS/MS, 141 *O*-GlcNAc sites were identified from component proteins in HeLa mitotic spindles and midbodies [109] and 458 *O*-GlcNAc sites in 195 proteins from mouse cerebrocortical brain tissue [108].

Moreover, the chemoenzymatic labeling approach can be readily coupled with BEMAD and CID-MS/MS for *O*-GlcNAc site mapping [111-114]. In one study, 35 *O*-GlcNAc sites corresponding to 25 *O*-GlcNAcylated proteins were identified from erythrocytes [114]. In addition, with the further integration of isobaric tag for relative and absolute quantitation technique (iTRAQ), the relative occupancy ratio between normal and diabetic erythrocytes was determined, revealing different *O*-GlcNAcylation at individual sites on proteins under distinct cellular conditions.

#### Metabolic labeling

In comparison to the enrichment methods mentioned above, which are performed in vitro, metabolic labeling offers an in vivo way to place a chemical handle onto *O*-GlcNAc proteins. This approach is based on the utilization of *N*-azidoacetylglucosamine (GlcNAz), an analog to GlcNAc. Studies have shown that GlcNAz can be tolerated by enzymes in the GlcNAc salvage pathway generating UDP-GlcNAz, which can finally be accepted by OGT and transferred to substrate proteins in living cells [115]. Therefore, by feeding cells with an appropriate amount of peracetylated GlcNAz, proteins initially modified by *O*-GlcNAc will be substituted with GlcNAz. Peracetylation allows the compounds to enter the cells and endogenous deacetylases rapidly remove the acetyl groups. GalNAz may also be used for labeling *O*-GlcNAc modified proteins, because it is readily epimerized to GlcNAz [116]. The GlcNAz-tagged proteins can be chemoselectively conjugated with a biotinylated phosphine reagent or a biotinylated alkyne reagent *via* Staudinger ligation [115,116] or click chemistry [117-120], respectively. After streptavidin-conjugated beads enrichment, tagged proteins are then digested, with the digests identified by MS. Recently, with this method, 185 *O*-GlcNAc sites were assigned to 80 proteins in HEK293 cells [120].

In other studies, the alkynyl-modified GlcNAc analog (GlcNAlk) has been exploited as a chemical reporter of *O*-GlcNAc modification in living cells [119,121]. In combination with click chemistry (with an azide-biotin reagent), streptavidin-conjugated beads enrichment, proteolytic digestion and MS, 374 putative *O*-GlcNAc proteins were identified [121]. One feature of GlcNAlk labeling is that, while GlcNAz can be metabolically interconverted to GalNAz [116,122], GlcNAlk does not, suggesting it may be a more specific metabolic reporter of *O*-GlcNAc modification.

Collectively, metabolic labeling has shown some advantages for facile enrichment of *O*-GlcNAc proteins. However, the major downside is that the cell’s enzymes prefer the natural substrate over the non-canonical ones, resulting in relatively low levels of tagging.

#### Quantification of O-GlcNAcylation

Global quantitative analysis of the levels of proteins and their *O*-GlcNAc sites is key to a systematic understanding of the molecular function of *O*-GlcNAc proteins in various biological processes. The traditional quantitation approach, which relies on high-resolution protein separation by 2-D gels and mass spectrometry identification of certain significantly altered spots, has been used for probing changes of *O*-GlcNAc proteins from several cell lines and tissues [75-78]. However, inherent drawbacks of the 2-D gel separation technique hinder its application for in-depth comparative analysis, as mentioned above. In contrast, the integration of stable isotope labeling with gel-free separation, specific enrichment, and mass spectrometry detection has been demonstrated to be a very powerful tool to provide quantitative information about *O*-GlcNAc changes between samples in control, diseases, and drug-perturbation conditions. There are mainly two ways: in vivo metabolic labeling and in vitro chemical reaction, to incorporate stable isotopes into *O*-GlcNAc proteins/peptides for mass spectrometry based quantification.

#### In vivo metabolic labeling-based O-GlcNAc quantification

As an in vivo approach, stable isotope labeling with amino acids in cell culture (SILAC) allows proteins to be labeled by growing cells in media containing isotopically labeled amino acids (e.g., ^13^C/^15^ N-arginine, ^13^C/^15^ N-lysine, ^13^C/^2^H-methionine). Due to the high quantification accuracy, SILAC has become a versatile tool for multiple proteomic applications [123-125]. Wang *et al.* evaluated *O*-GlcNAc proteomic changes upon stimulation of cells by lithium, a selective inhibitor to glycogen synthase kinase-3 (GSK-3) which is extensively involved in many signaling pathways [81]. By combining SILAC, CTD 110.6-bound beads enrichment, and LC-MS/MS, they identified 45 potentially *O*-GlcNAcylated proteins, 10 of which showed increased *O*-GlcNAcylation while 19 showed decreased *O*-GlcNAcylation upon GSK-3 inhibition [81]. Their results indicate a complex interplay between phosphorylation and *O*-GlcNAcylation within signaling networks. With a similar approach, Zachara *et al.* investigated the changes of *O*-GlcNAcylated proteins of cells in response to heat shock [82]. Amongst the proteins identified, some DNA-binding proteins showed elevated levels of O-GlcNAcylation, suggesting a role for *O*-GlcNAc in regulating DNA damage signaling or repair. In another study, by using a combination of SILAC, chemoenzymatic labeling-based enrichment, and LC-MS/MS, altered phosphorylation of key proteins in cellular midbodies was revealed upon the over-expression of OGT [109], further illustrating the intricate crosstalk between O-GlcNAcylation and phosphorylation of proteins in the regulation of cell division.

Even though SILAC has been demonstrated to be a powerful tool in quantitative proteomic studies for cultured cells, it is still not very practical for analyzing biological samples that can not be grown in culture, such as tissues or body fluids. The further development of SILAC techniques (i.e., tissues and even whole animal-targeted SILAC [126,127]) should further benefit related applications including quantitative *O*-GlcNAc profiling.

#### In vitro labeling-based O-GlcNAc quantification

As a non-biased approach, the in vitro labeling involves incorporation of stable isotopic tags onto selective sites on proteins/peptides *via* chemical reactions. Isotopic labeling can be introduced at the N-/C-terminus, on specific amino acid residues (e.g. cysteine [128]), or at the C-terminus of peptides during trypsin-catalyzed-^18^O labeling of proteins [129]. Amongst those techniques, N-terminus-targeted labeling, especially the isobaric tags for relative and absolute quantitation (iTRAQ) [130] and isotope dimethyl labeling [131,132], has been adopted for *O*-GlcNAc quantification. In one study, iTRAQ was coupled with the chemoenzymatic labeling enrichment and LC-MS/MS to investigate the extent of *O*-GlcNAcylation on human erythrocyte proteins from diabetic and normal individuals [114]. Twenty-five *O*-GlcNAcylated erythrocyte proteins were identified with differential *O*-GlcNAcylation level between diabetic and normal erythrocytes, suggesting a potential regulatory role of *O*-GlcNAcylation on erythrocyte proteins in response to glycemic status. In another study, isotope dimethyl labeling was used with chemoenzymatic labeling enrichment and LC-MS/MS for probing the dynamics of *O*-GlcNAcylation in brain [105]. Differential *O*-GlcNAcylation on several proteins involved in the regulation of transcription and mRNA translocation was revealed, suggesting important roles of protein *O*-GlcNAcylation in mediating the communication between neurons. As a quite different approach, BEMAD can introduce isotopic labels (i.e., deuterated DTT) onto originally *O*-GlcNAc modified Ser/Thr residues prior to the thio-affinity enrichment [96], allowing the evaluation of site-specific *O*-GlcNAc changes. Moreover, by normalizing the level of site specific *O*-GlcNAc peptides to that of the corresponding proteins, relative site occupancy ratio (ROR) between different biological contexts can be obtained. By comparing iTRAQ based protein quantification and isotopic DTT-mediated BEMAD-based *O*-GlcNAc peptide quantification, the *O*-GlcNAc site occupancy on erythrocyte proteins from diabetic and normal individuals was determined [114]. Of note, certain proteins with significant *O*-GlcNAc site occupancy changes may serve as a sensitive diagnostic tool for the early detection of diabetes.

#### Label-free quantification approaches

There has been increasing interest in the development of label-free mass-spectrometry quantification techniques, due to the potential limitations of the isotopic labeling-based quantification approaches (e.g., increased complexity of sample preparation, high cost of the reagents, and incomplete labeling). One label-free approach is based on the comparison of the peptide peak intensity or spectral count [133], the applicability of which is still to be explored for *O*-GlcNAc quantification though. Multiple reaction monitoring (MRM) or selected reaction monitoring (SRM), a non-scanning technique primarily on triple-quadrupole mass spectrometers, provides another promising tool for the quantification of target proteins [134]. Recently, MRM-MS has been applied to quantify a standard *O*-GlcNAcylated peptide down to 3 fmol and then monitor the increased *O*-GlcNAcylation of several peptides of GSK-3β in human embryonic stem cells upon the treatment with an *O*-GlcNAcase inhibitor [135].

Without doubt, by integrating sophisticated quantification procedures with increasingly efficient enrichment methods and advanced MS techniques, *O*-GlcNAcomic profiling is foreseeable in the near future, which would facilitate the in-depth elucidation of the important roles of protein *O*-GlcNAcylation in diverse biological contexts.

### Protein microarray-based O-GlcNAcomics

Distinct from MS, protein microarray represents another high-throughput method for the analysis of PTMs such as phosphorylation and *N*-glycosylation [136]. Tarrant *et al.* used a protein array to screen for protein substrates of *O*-GlcNAcylated and/or phosphorylated CKII [43]. Their results reveal that the substrate spectrum changes after binding to its interacting partner Pin1 and that the substrate selectivity of CKII is delicately modulated by *O*-GlcNAcylation and phosphorylation. To identify protein kinases that are potentially *O*-GlcNAcylated, Dias and coworkers used a functional human protein array containing 152 kinases as a substrate for OGT in vitro. Intriguingly, they identified 42 kinases that are *O*-GlcNAcylated in vitro (~39% of all the kinases analyzed) [137], suggesting that a number of protein kinases may be regulated by *O*-GlcNAcylation and this regulation may further complicate the already intricate relationship between *O*-GlcNAcylation and phosphorylation. Indeed, recent studies have shown that a number of important kinases (including CKII [43], CaMKIV [138], PKC [139], Akt [140], I*κ*B kinase [141], among others) are regulated by *O*-GlcNAcylation. With the further optimization and improvement of related techniques, protein microarrays will still be a valuable technology for *O*-GlcNAcomic studies.

## Conclusions and perspectives

Over the first two decades since its discovery, *O*-GlcNAcylation was determined to be on ~500 proteins [142]. With the introduction of new enrichment techniques and advanced mass spectrometers, the number of *O*-GlcNAcylated proteins has been increased to >4000 (a detailed list is being compiled). More importantly, numerous *O*-GlcNAc sites have also been mapped, which not only significantly facilitate deciphering the crucial roles of *O*-GlcNAc on individual proteins in various biological processes, but also provide us a much deeper insight on how this modification closely interplays with many other PTMs (especially phosphorylation) in complex molecular networks.

**Figure 2:**
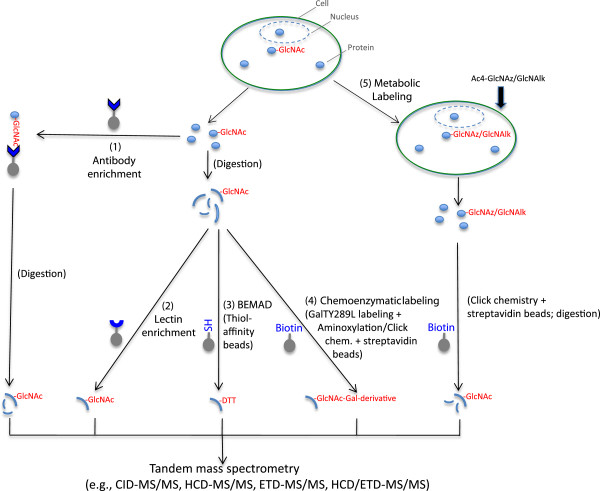
**Scheme for the enrichment of *O*-GlcNAcylated proteins/peptides.** Most commonly used strategies with antibody enrichment **(1)**, lectin enrichment **(2)**, BEMAD **(3)**, chemoenzymatic labeling **(4)** and metabolic labeling **(5)** are illustrated. In (1), proteins are captured onto antibody/antibodies-conjugated beads, and the enriched ones are digested and identified by tandem mass spectrometry. In (2), (3), and (4), proteins are digested into peptides, which are captured with lectin-conjugated resin (2), thio-capture column after BEMAD (3), and streptavidin-conjugated beads after chemoenzymatic labeling (4), with the enriched peptides identified by tandem mass spectrometry. In (5), cells are fed with GlcNAc analogs GlcNAz and GlcNAlk, and the GlcNAz- and GlcNAlk-containing proteins are subjected to click chemistry, streptavidin-conjugated beads enrichment and digestion, with the digests analyzed by tandem mass spectrometry. Note: The cocktail usage of several methods (e.g., chemo-enzymatic/metabolic labeling and BEMAD) has also been applied in some cases.

However, we are still in the early stage of *O*-GlcNAc profiling, compared to the rapidly maturing characterization of other PTMs (e.g., phosphorylation, *N*-glycosylation, lysine acetylation, and ubiquitination) for which a handful of highly efficient and robust tools are available. Although many enrichment methods have been developed for *O*-GlcNAc proteins/peptides (Figure 2), they are still far from being applicable routinely to the analysis of samples, especially for complex ones when large-scale comparative *O*-GlcNAcomic profiling is desired. Moreover, the newly designed mass spectrometers (especially the ETD-equipped ones) are not widely available to most labs, which hampers the site-oriented *O*-GlcNAc functional assays. In addition, there are limited software and algorithms specifically designed for *O*-GlcNAc site prediction as well as mass spectrometry data mining.

Considering the extremely important roles *O*-GlcNAc plays, the complete repertoire of *O*-GlcNAcylated proteins as well as their specific sites must be defined. To this end, several aspects about improving *O*-GlcNAc profiling are anticipated. 1) Refinement of current enrichment techniques and development of novel ones should still be a topic of intense interest. 2) How to make full use of the capacity and improve the performance of mass spectrometers for *O*-GlcNAc detection remains to be addressed. The combination of different fragmentation modes (e.g., HCD plus ETD) would be a powerful tool for improved *O*-GlcNAc identification and site mapping. Moreover, the potential for ETD in applications, such as multiple reaction monitoring (MRM) for *O*-GlcNAc peptides and top-down characterization for *O*-GlcNAc proteins, should be explored. 3) Quantitative proteomic techniques should be further adopted in more *O*-GlcNAc studies. 4) Designing novel bioinformatic tools for *O*-GlcNAc research will be another goal in the future. 5) The development of a large number of site-specific antibodies, as are now available for protein phosphorylation, will be critical to the rapid advancement of this field by biologists. Taken together, as with other PTMs, technology integration will hasten the maturation of diverse methods for *O*-GlcNAc profiling. We are sure that the technology-driven *O*-GlcNAcomics will boom soon, which would profoundly contribute to the elucidation of crucial functions of protein *O*-GlcNAcylation in versatile physiological and pathological conditions and to a systems perspective of molecular mechanisms in biological networks.

## Abbreviations

*O*-GlcNAc: *O*-linked β-D-*N*-acetylglucosamine; *O*-GlcNAcylation: *O*-linked β-D-*N*-acetylglucosamine addition; UDP-GlcNAc: Uridine diphospho-*N*-acetylgluco-samine; OGT: *O*-GlcNAc transferase; *O*-GlcNAcase: β-*N*-acetyl-glucosaminidase; PTM: Post-translational modification; PNGase F: Peptide: *N*-glycosidase F; GalT: β1-4-galactosyltransferase; SDS-PAGE: SDS-polyacrylamide gel electrophoresis; HPLC: High performance liquid chromatography; MS: Mass spectrometry; CID: Collision induced dissociation; HCD: High-energy collision dissociation; ETD: Electron transfer dissociation; WGA: Wheat germ agglutinin; BEMAD: Beta elimination/Michael addition with dithiothreitol; SILAC: Stable isotope labeling of amino acids in cell culture; iTRAQ: Isobaric tag for relative and absolute quantitation.

## Competing interests

Original research in the author’s laboratory is supported by NIH R01CA42486, R01DK61671, N01-HV-00240, P01HL107153, and the Patrick C. Walsh Prostate Cancer Research Fund. Dr. Hart receives a share of royalty received by the university on sales of the CTD 110.6 antibody, which are managed by JHU. The authors have no other relevant affiliations or financial involvement with any organization or entity with a financial interest in or financial conflict with the subject matter or materials discussed in the manuscript apart from those disclosed.

## Authors' contributions

JM drafted the manuscript. Both authors edited and approved the final manuscript.
